# Trend shift in proximal humeral fracture treatment: a detailed review of national shoulder arthroplasty data

**DOI:** 10.55730/1300-0144.5884

**Published:** 2024-09-07

**Authors:** İbrahim BOZKURT, İbrahim KAYA, Umut ÖKTEM, Sinan YILMAZ, Naim ATA, Mustafa Mahir ÜLGÜ, Şuayip BİRİNCİ, Fatih KARAASLAN, İzzet BİNGÖL, Durmuş Ali ÖÇGÜDER

**Affiliations:** 1 Department of Orthopaedics and Traumatology, Ankara City Hospital, Ankara, Turkiye; 2 Department of Orthopaedics and Traumatology, Dr. Abdurrahman Yurtaslan Ankara Oncology Training and Research Hospital, Ankara, Turkiye; 3 Department of Orthopaedics and Traumatology, Memorial Ankara Hospital, Ankara, Turkiye; 4 Ministry of Health, General Directorate of Health Information Systems, Ankara, Turkiye; 5 Ministry of Health, Deputy Minister, Ankara, Turkiye; 6 Department of Orthopaedics and Traumatology, Memorial Kayseri Hospital, Kayseri, Turkiye; 7 Department of Orthopaedics and Traumatology, Ankara Yıldırım Beyazıt University, Ankara, Turkiye

**Keywords:** Proximal humerus fracture, hemiarthroplasty, reverse shoulder arthroplasty, total shoulder arthroplasty, national trends

## Abstract

**Background/aim:**

This study aimed to scrutinize nationwide utilization trends of shoulder arthroplasty for proximal humerus fractures (PHFs) using a comprehensive national surgical database.

**Materials and methods:**

A retrospective study was conducted with 4181 patients who underwent shoulder arthroplasty due to PHF between 2016 and 2022 using national health records. They are grouped as hemiarthroplasty (HA), anatomical total shoulder arthroplasty (TSA), and reverse shoulder arthroplasty (RSA). The patients’ demographic data, length of hospital stay, revision histories, transfusion rates, mortality data, trends in arthroplasty methods over the years, the distribution of cases by hospital characteristics and geographical regions were analyzed.

**Results:**

Treatment with HA was administered to 22.1% of patients, TSA to 30.2%, and RSA to 47.7%. The lowest revision rate was observed after HA (4.3%), while higher rates were recorded after TSA (7.9%) and RSA (7.4%) (p = 0.019). It was observed that there was a significant increasing trend in RSA rates and a decreasing trend in HA and TSA rates over time (p < 0.001).

**Conclusion:**

From 2016 to 2022, there appears to have been a significant increase in the utilization of RSA for the arthroplasty treatment of proximal humeral fractures in Türkiye, and it is used more frequently than HA. However, revision rates after RSA are still higher than those after HA.

**Level of evidence:**

Level III, retrospective cohort study.

## 1. Introduction

Proximal humerus fracture (PHF) is one of the most common osteoporotic fractures in older people, accounting for approximately 4% to 5% of all fractures [1]. Various treatment options are available for PHFs, including nonsurgical treatment, osteosynthesis, and arthroplasty [2]. Most PHFs can be treated conservatively. The decision regarding surgery depends on patient-related factors such as age, functional capacity, comorbidities, and the type of fracture [3]. While open reduction and internal fixation (ORIF) is traditionally used for the surgical treatment of PHFs, these fractures are characterized by comminution of osteopenic bone and fracture fragments in the elderly population, thus leading to conventional osteosynthesis techniques being less successful [4–7].

Anatomical total shoulder arthroplasty (TSA) or hemiarthroplasty (HA) is used to treat four-part displaced fractures or fracture-dislocations of the proximal humerus. HA is more commonly used than TSA, except in patients with degenerative arthritis. Reverse shoulder arthroplasty (RSA) is a good option for the treatment of PHFs in elderly patients with significant rotator cuff dysfunction or inability to achieve tuberosity osteosynthesis [8–11]. Although HA was the arthroplasty option used most frequently in the past, the popularity of RSA has been increasing in recent years [9,12,13].

Currently, the absence of definitive guidelines for selecting indications and treatment procedures for patients with PHFs results in substantial variability in surgical practices. This study is designed to scrutinize nationwide trends in the utilization of shoulder arthroplasty for PHFs using a comprehensive national surgical database. Our objectives are to identify any temporal changes and to compare the characteristics and revision rates among patients treated with HA, anatomical TSA, and RSA.

## 2. Materials and methods

A retrospective study was conducted using health records from the National Personal Health Record System of Türkiye (Turkish Ministry of Health) for individuals aged 18 and over who presented to public, private, and university health institutions between 2016 and 2022 [14]. A significant amount of big data is available, as this system includes all healthcare system data. The register contains personal identification numbers, patient age, sex, residence, length of hospital stay, primary and secondary diagnoses, and surgical procedures performed during hospital stay. The study was conducted in compliance with the Declaration of Helsinki and received approval from the Turkish Ministry of Health, which included a waiver for informed consent for retrospective data analysis, in accordance with the health information privacy law (ID: 95741342-020/27112019).

The database was searched using international classification codes in accordance with the World Health Organization’s 10th Revision of the International Statistical Classification of Diseases and Related Health Problems (ICD-10). The ICD code S42.2 (code for closed PHFs) was used to identify patients older than 18 years who had sustained a PHF between January 2016 and December 2022. Patients with open fractures, multitrauma, or pathological fractures; those who had undergone osteosynthesis, and those with missing data were excluded from the study. Only cases of PHFs treated surgically were examined, as most conservatively treated patients could not be accurately identified in the data system. Subsequently, patients who had undergone surgery were extracted from the e-health database using operation-specific procedure codes, implant-specific codes from invoices, and treatment-specific codes.^1^ As a result, 4181 proximal humerus fractures that underwent shoulder arthroplasty were included in the study. After these patients were identified, they were grouped into subsets of HA (612320), TSA (612550), and RSA (612551) according to the procedure codes used. For each of the three arthroplasty groups, separate procedure codes and implant-specific codes are systematically defined, and the code corresponding to the procedure applied must be entered into the system in order to invoice the procedure. The demographic data of the patients—including age, sex, length of hospital stay, revision history, time between primary and revision surgery, transfusion rates, in-hospital mortality data, trends in arthroplasty methods from January 2016 to December 2022, distribution of cases according to hospital characteristics (private hospitals, community hospitals [2nd level state and 3rd level state training and research hospital], and university hospitals), and geographical regions (there are 7 geographical regions in Türkiye)—were analyzed from the e-health database. All arthroplasty patients with a minimum follow-up period of at least 2 years (from 2016 to 2021) were reevaluated and included in the revision analysis. Data were collected by screening the procedure codes used for revision surgery (revision arthroplasty and prosthesis removal).

### 2.1. Statistical analysis

SPSS 25 (IBM Corporation, Armonk, NY, USA) was used for statistical analysis. Version 2023.03.0 of R Studio was employed for mapping, utilizing the following packages: “devtools v2.4.5,” “TRmaps v0.0.0.900,” “ggplot2 v3.4.2,” “scales v1.2.1,” “dplyr v1.0.10,” and “sf v1.0-12.” Mean, standard deviation, frequency, and percentage were used as descriptive statistics. Chi-squared tests (both Pearson and likelihood ratio) were used for categorical variables, while the Kruskal–Wallis test was utilized for continuous variables. The significance level was set at 0.05 for all tests.

## 3. Results

**Figure 1:**
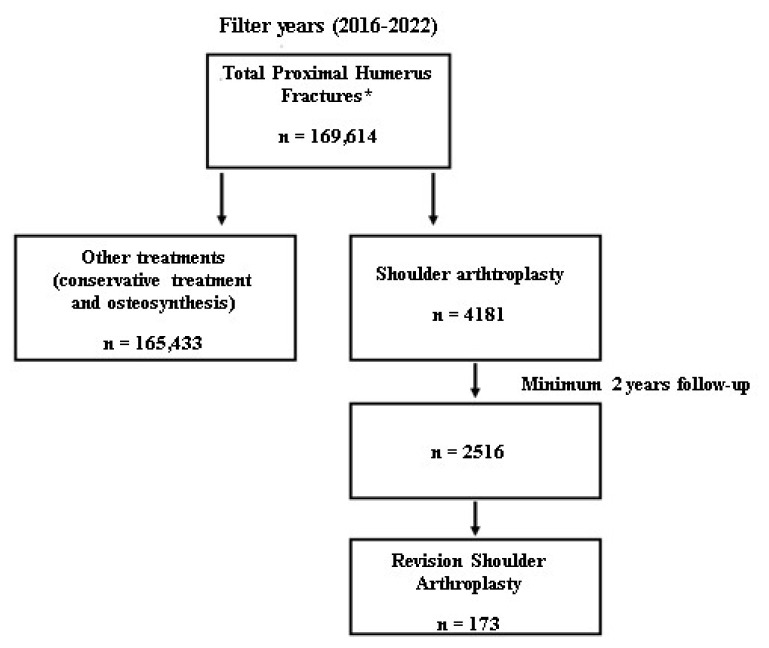
Flow diagram of patients with proximal humeral fractures and treated with shoulder arthroplasty, identified between 2016 and 2022. * The ICD code: S42.2 (code for closed proximal humerus fractures).

Between 2016 and 2022, a total of 169,614 patients with PHFs were identified using ICD-10 codes. A total of 4181 shoulder arthroplasty cases were included in the study (Figure 1). The mean age of these patients was 73.2 ± 10.8 (range: 19–102); 25.3% were male, and 79.7% were over 65 years of age. Of the arthroplasty patients that presented for treatment of PHFs, 22.1% (n = 922) were treated with HA, 30.2% (n = 1264) with TSA, and 47.7% (n = 1995) with RSA. The ages of the patients were homogeneous in terms of the treatment methods (p = 0.096). It was concluded that RSA was predominantly applied to women (49.3%), while HA (25.3%) and TSA (31.8%) were more frequently applied to men (p < 0.001). Blood transfusion was administered to 1502 (35.9%) of the 4181 patients. The distribution of transfused patients was as follows: 23% in the HA group, 31.9% in the TSA group, and 45.1% in the RSA group. Additionally, the rate of transfusion was 37.5% in the HA group, 37.9% in the TSA group, and 33.9% in the RSA group. The transfusion rate was lowest in the patients who had undergone RSA (p = 0.037). Moreover, 1995 (47.7%) patients had undergone surgery at community hospitals, 864 (20.7%) at university hospitals, and 1322 (31.6%) at private hospitals. RSA was the method most commonly used (51.8%) in university hospitals (p < 0.001) (Table 1).

Considering the geographical regional distribution of cases; RSA was applied most in Central Anatolia Region (56.6%) and least in Eastern Anatolia Region (22.5%), TSA was used most in Eastern Anatolia Region (57.5%) and least in the Aegean Region (25.1%), and HA was used most in Southeastern Anatolia Region (30.3%) and least in Central Anatolia Region (16.9%) (p < 0.001) (Table 2).

In order to evaluate the revision rates, arthroplasty patients with a minimum follow-up of 2 years were evaluated. Revision surgery was performed in 173 (6.9%) of the 2516 arthroplasty patients included in the evaluation. When the revision status was evaluated internally for each method, HA had the lowest revision rate (24 patients, 4.3%), while the revision rates were higher for TSA (71 patients, 7.9%) and RSA (78 patients, 7.4%) (p = 0.019). Being male and young increased the likelihood of revision (p = 0.024, p < 0.001, respectively). Additionally, there was no variability in the revision rate according to years (p = 0.172) (Table 3). The revision surgery was performed, on average, 15.3 ± 15.5 months after the first operation. The average time was 13 ± 11 months in the HA group, 13.3 ± 14.2 months in the TSA group, and 17.7 ± 17.5 months in the RSA group (p = 0.224). The overall in-hospital mortality rate was 0.6%. Of these cases, eight (34.8%) were attributed to HA, nine (39.1%) to TSA, and six (26.1%) to RSA.

**Figure 2:**
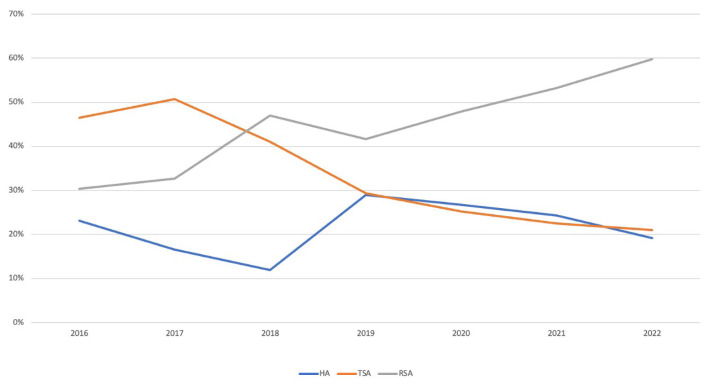
Change in methods over the years.

In the seven years studied, the rate of HA decreased from 23.1% to 19.2%, and the rate of TSA decreased from 46.5% to 21%. The rate of RSA increased from 30.4% to 59.8%. When comparing cases of arthroplasty due to a PHF between 2016 and 2022, there was a significant upward trend in the RSA rate over time, a decrease in the HA and TSA rates, and patients in 2022 were more likely to have undergone RSA compared to earlier years (p < 0.001) (Figure 2).

## 4. Discussion

In the present study, nationwide data were used to analyze surgical trends in patients undergoing arthroplasty for PHF from 2016 to 2022. The seven-year results indicated that the use of reverse shoulder prosthesis in Türkiye is higher than that of HA and TSA. Among the arthroplasty procedures, the utilization rate of RSA increased annually, while the utilization rate of HA decreased. In 2016, RSA accounted for 30.4% of all arthroplasty cases, while this rate increased to 59.8% in 2022. These findings are consistent with the current literature; similarly, in other population-based studies, the rate of RSA has increased significantly over the years compared to other arthroplasty procedures [15–20]. This trend towards reverse shoulder arthroplasty in this country in recent years aligns with global population arthroplasty trends for PHF. The changes in RSA utilization rates are likely related to the growing popularity of this technique as a reliable option for managing PHFs in the population aged over 65.

Although anatomical TSA is commonly performed for osteoarthritis, it is also a popular arthroplasty procedure in cases where a functional rotator cuff is present following a PHF [8,21]. According to data from the United States published by Schairer et al. [17], 51.3% of arthroplasty procedures for PHF in 2013 were HA, 45.1% were RSA, and 3.6% were TSA. In the current study, the rates of TSA are higher than those reported in the literature. Recent studies have increasingly demonstrated the functional outcomes of RSA are more favorable [22–25]. Therefore, we can conclude that the trend in Türkiye has shifted in alignment with this emerging trend in the literature.

In a retrospective review of 384,158 patients with PHFs between 2010 and 2019, those who underwent RSA were older and more likely to be female compared with the patients who underwent ORIF or HA [16]. In their database analysis covering the years 2009 to 2012, Rosas et al. reported that while the use of HA in patients aged over 65 years was higher than that of RSA, the preference for RSA increased with patient age [26]. In the current study, the age distribution was homogeneous among the HA, TSA, and RSA groups. Furthermore, the use of RSA was higher in females, whereas HA and TSA were more commonly performed in males.

The results of HA and RSA are frequently discussed in the literature. In a systematic review conducted by Mata-Fink et al. in 2013, the functional results of HA were found to be poor in cases of rotator cuff failure, while the functional results of RSA are superior to those of HA in the treatment of PHF in older adults [27]. According to a metaanalysis of 67 studies, RSA has lower revision rates compared to HA and provides improved function with better recovery of active advanced flexion and abduction [22]. Similarly, numerous studies have reported that the likelihood of success for HA is lower than that for RSA in elderly patients, attributed to factors such as tuberosity nonunion and rotator cuff failure [12,13,28,29]. In the present study, the clinical results were not available in the database; however, the existing literature indicates a national trend towards the use of RSA. A review of the literature reveals that new investigations have been conducted in response to instances of functional failure in patients who have undergone HA and RSA. Notably, RSA is gaining popularity as a successful alternative, particularly for conditions such as rotator cuff insufficiency. However, the data from this country indicate that the revision rates of HA are significantly lower, in contrast to those reported in the literature. This may be explained by the relatively low functional expectations and sociocultural level of the patients, leading them to more readily accept the results of the surgery.

Another aim of the present study was to evaluate the rates of revision arthroplasty among HA, TSA, and RSA. The overall revision rate of arthroplasty cases with a minimum follow-up period of 2 years was 6.9%, while HA had the lowest revision rate and TSA had the highest revision rate. The time to revision after the initial surgery was comparable across all three groups. In a national database analysis by Alrabaa et al., the revision arthroplasty rate for RSA was 3.9%, while the rate of implant removal and spacer insertion was 4.3% [16]. In systematic reviews from 2013 and 2015 comparing HA and RSA in the treatment of PHF, revision rates were reported as 6.4% and 4% for HA and 1.3% and 0.9% for RSA, respectively—results that differ from those observed in our study [10,30].

In a similar study evaluating patients from a national database in the United States between 2000 and 2013, the mean hospital stay was slightly shorter in the RSA group (3.9 days) than in the HA group (4.1 days). In terms of in-hospital mortality, there was no significant difference between the HA (0.26%) and RSA (0.44%) groups [17]. Sabesan et al. reported no significant difference in in-hospital mortality rates between HA and RSA, with rates of 0.5% and 0.6%, respectively [20]. In the current study, the overall in-hospital mortality rate was 0.6%, with rates of 0.87% in the HA group, 0.71% in the TSA group, and 0.3% in the RSA group.

In a study involving 542 patients who underwent surgical treatment for PHFs, the overall blood transfusion rate after arthroplasty was 27.2% [31]. In similar studies, the transfusion rates following postfracture arthroplasty procedures were reported as 18.4% and 32.7% [32,33]. Similar to our study, Jeong et al. reported a transfusion rate of 30.8% in HA cases after fracture [34]. In a study conducted in 2016 involving 1792 patients from the American College of Surgeons database, the transfusion rate was 15.6% among HA patients due to PHF and 20% among RSA/TSA patients [35]. In the current study, the overall transfusion rate was 35.9%, with rates of 37.5% in the HA group, 37.9% in the TSA group, and 33.9% in the RSA group, slightly higher than those reported in the literature.

This study has several limitations. First, fracture types could not be analyzed separately because it utilized a national database study, which evaluated all PHFs collectively. Second, due to the absence of information on clinical outcomes in the database, it was not possible to compare these outcomes between the arthroplasty procedures. Additionally, as this was a multicenter study, it included potential variations in surgical techniques. Finally, since diagnostic and procedural codes are submitted by practitioners and coders, there is a risk of human error in the coding and billing processes.

While the data suggests a shift in preference from HA to RSA for PHF treatment in Türkiye from 2016 to 2022, it is crucial to understand the underlying factors driving this trend. The increasing popularity of RSA may be attributed to its advantages in specific patient populations, such as those with significant rotator cuff dysfunction or difficulties in achieving tuberosity osteosynthesis.

However, the persistently higher revision rates of RSA compared to HA require a deeper investigation into potential improvements in surgical techniques, postoperative care, and patient selection criteria. Further research is needed to optimize the decision-making process in the choice of arthroplasty methods for PHFs, ultimately aiming to improve patient outcomes and reduce revision rates.

### 4.1. Highlights

- In Türkiye, the approach to treating proximal humerus fractures has shifted over the years from HA to RSA.
- The increasing popularity of RSA may be attributed to its advantages in specific patient populations.
- However, revision rates after RSA remain higher than those observed after HA.

## Figures and Tables

**Table 1:** Analysis results based on the performed operations.

Variables	Categories	HA (n = 922)	TSA (n = 1264)	RSA (n = 1995)	p
Sex	Male (n = 1056)	267 (25.3)	336 (31.8)	453 (42.9)	<0.001
	Female (n = 3125)	655 (21.0)	928 (29.7)	1542 (49.3)	
Age		73.8 ± 11.5	73.6 ± 11.4	73.03 ± 10.1	0.096
Transfusion	No (n = 2679)	576 (21.5)	785 (29.3)	1318 (49.2)	0.037
	Yes (n = 1502)	346 (23)	479 (31.9)	677 (45.1)	
Hospital volume	University (n = 864)	170 (19.7)	247 (28.5)	447 (51.8)	<0.001
	Community (n = 1995)	372 (18.6)	735 (36.8)	888 (44.5)	
	Private (n = 1322)	379 (28.7)	283 (21.4)	660 (49.9)	

Continuous variables were expressed as mean ± standard deviation and categorical variables were expressed as frequency (percentage).

**Table 2:** Geographical regional distribution of cases.

Geographical regions	HA	TSA	RSA	p
Marmara	303 (22.2)	371 (27.2)	688 (50.5)	<0.001
Aegean	223 (25.3)	222 (25.1)	438 (49.6)	
Mediterranean	129 (23.5)	234 (42.5)	187 (34.0)	
Central Anatolia	152 (16.9)	239 (26.5)	510 (56.6)	
Black Sea	87 (23.0)	149 (39.3)	143 (37.7)	
Eastern Anatolia	8 (20.0)	23 (57.5)	9 (22.5)	
Southeastern Anatolia	20 (30.3)	26 (39.4)	20 (30.3)	

Categorical variables were expressed as frequency (percentage).

**Table 3:** Comparison of revision status based on variables.

Variables	Categories	No revision	Revision	p
Method	HA (n = 563)	539 (97.5)	24 (4.3)	0.019
	TSA (n = 903)	832 (92.1)	71 (7.9)	
	RSA (n = 1050)	972 (92.6)	78 (7.4)	
Sex	Male (n = 647)	590 (91.2)	57 (8.8)	0.024
	Female (n =1869)	1753 (93.8)	116 (6.2)	
Age		74.8 ± 10.8	69.7 ± 10.7	<0.001
Years	2016 (n = 273)	250 (91.6)	23 (8.4)	0.172
	2017 (n = 398)	362 (91.0)	36 (9.0)	
	2018 (n = 504)	475 (94.2)	29 (5.8)	
	2019 (n = 684)	636 (93.0)	48 (7.0)	
	2020 (n = 657)	620 (94.4)	37 (5.6)	

Continuous variables were expressed as mean ± standard deviation and categorical variables were expressed as frequency (percentage).

## Footnotes

1 Türkiye Cumhuriyeti Sağlık Bakanlığı (2019). Sağlık Kodlama Referans Sunucusu (SKRS) [online]. Website https://skrs.saglik.gov.tr/ [accessed 06 May 2024] (in Turkish).
